# Putative roles of SLC7A5 (LAT1) transporter in pain

**DOI:** 10.1016/j.ynpai.2020.100050

**Published:** 2020-06-30

**Authors:** Sascha R.A. Alles, Kimberly Gomez, Aubin Moutal, Rajesh Khanna

**Affiliations:** a Department of Anesthesiology and Critical Care Medicine, University of New Mexico School of Medicine, United States; b Department of Pharmacology, University of Arizona, United States; c Department of Anesthesiology, College of Medicine, The University of Arizona, Tucson, AZ 85724, United States; d BIO5 Institute, University of Arizona, 1657 East Helen Street Tucson, AZ 85719, United States; e Center for Innovation in Brain Sciences, University of Arizona, Tucson, AZ 85721, United States; f Regulonix Holding Inc., Tucson, AZ, United States

**Keywords:** LAT1, Inflammation, Neuropathic pain, mTOR, Ion channels, Gabapentinoids

## Abstract

Large amino acid transporter 1 (LAT1), also known as SLC7A5, is an essential amino acid transporter that forms a heterodimeric complex with the glycoprotein cell-surface antigen heavy chain (4F2hc (CD98, SLC3A2)). Within nociceptive pathways, LAT1 is expressed in the dorsal root ganglia and spinal cord. Although LAT1 expression is upregulated following spinal cord injury, little is known about LAT1 in neuropathic pain. To date, only circumstantial evidence supports LAT1/4F2hc’s role in pain. Notably, LAT1′s expression and regulation link it to key cell types and pathways implicated in pain. Transcriptional regulation of LAT1 expression occurs via the Wnt/frizzled/β-catenin signal transduction pathway, which has been shown to be involved in chronic pain. The LAT1/4F2hc complex may also be involved in pain pathways related to T- and B-cells. LAT1′s expression induces activation of the mammalian target of rapamycin (mTOR) signaling axis, which is involved in inflammation and neuropathic pain. Similarly, hypoxia and cancer induce activation of hypoxia-inducible factor 2 alpha, promoting not only LAT1′s expression but also mTORC1′s activation. Perhaps the strongest evidence linking LAT1 to pain is its interactions with key voltage-gated ion channels connected to nociception, namely the voltage-gated potassium channels Kv1.1 and Kv1.2 and the voltage-gated sodium channel Nav1.7. Through functional regulation of these channels, LAT1 may play a role in governing the excitatory to inhibitory ratio which is altered in chronic neuropathic pain states. Remarkably, the most direct role for LAT1 in pain is to mediate the influx of gabapentin and pregabalin, two first-line neuropathic pain drugs, that indirectly inhibit high voltage-activated calcium channel auxiliary subunit α2δ-1. In this review, we discuss the expression, regulation, relevant signaling pathways, and protein interactions of LAT1 that may link it to the development and/or maintenance of pain. We hypothesize that LAT1 expressed in nociceptive pathways may be a viable new target in pain.

## Highlights

• Large amino acid transporter 1 (LAT1, SLC7A5), is an essential amino acid transporter.
• Expressed in the DRG and spinal cord, circumstantial evidence supports a tenous link for LAT1 in pain.
• LAT1 binds to voltage-gated ion channels involved in pain.
• LAT1 mediates the influx of gabapentin and pregabalin, two first-line neuropathic pain drugs.
• We hypothesize that LAT1 expressed in nociceptive pathways may be a viable new target in pain.

## 1 Introduction

Large amino acid transporters (LATs) import essential amino acids into cells. There are four types of LATs, also known as Solute Carrier Family 7 Member 5 (SLC7A5 or LAT1), SLC7A8 (LAT2), SLC43A1 (LAT3), and SLC43A2 (LAT4) (Wang and Holst, 2015). The most abundant, LAT1, is a Na^+^-independent exchanger of large neutral amino acids (Kanai et al., 1998, Segawa et al., 1999). It is linked by a disulfide bond (Kanai et al., 1998, Verrey et al., 2000) and polar interactions (Yan et al., 2019) to 4F2 cell-surface antigen heavy chain (4F2hc), also known as SLC3A2. 4F2hc is a glycoprotein that allows the formation of stable transporter complexes for their localization to the plasma membrane where they are functional (Kanai et al., 1998, Yan et al., 2019). LAT1 is an antiporter that participates in the selective transport at the blood brain barrier (Boado et al., 1999, Kido et al., 2001). It is expressed in various tissues, including the brain (Kageyama et al., 2000, Matsuo et al., 2000), retina (Tomi et al., 2005), cornea (Jain-Vakkalagadda et al., 2003), colon (Fraga et al., 2005). LAT1 is strongly expressed in malignant tumors presumably to support their continuous growth and proliferation (Yanagida et al., 2001). In addition to transporting L-leucine, L-phenylalanine, L-histidine, and L-tryptophan, LAT1 also recognizes thyroid hormones (Friesema et al., 2001) and some pharmaceutical compounds such as L-DOPA (Kageyama et al., 2000), 2-(1-(aminomethyl)-cyclohexyl)acetic acid (gabapentin; GBP) (Dickens et al., 2013), 3-(aminomethyl)-5‑methyl-hexanoic acid (pregabalin; PGB) (Takahashi et al., 2018), among others. LAT1 only serves as a transporter for a handful of drugs, which makes it relatively unique to gabapentinoid pain therapeutics and their mechanism of action. Global LAT1 knockout mice were found to be embryonically lethal (Poncet et al., 2014), which could be due to its essential activity in cells of importing large neutral amino acids. In the sensory system, the best known role of LAT1 is to mediate the influx of GBP and PGB (Dickens et al., 2013, Takahashi et al., 2018), which are two front-line medications for management of neuropathic pain (see gabapentinoid transport section). Therefore, in this review we will address the expression, regulation, function and relevant interactions of this transporter that link it to the onset, development and/or maintenance of pain.

## 2 Expression of LAT1 in nociceptive pathways

**Fig. 1:**
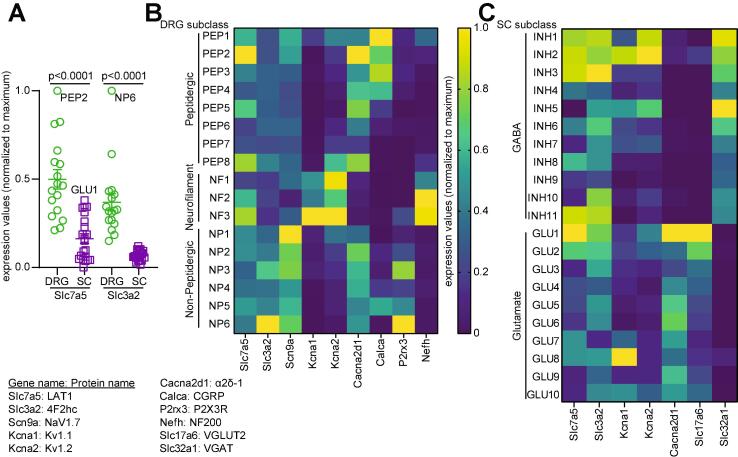
E**xpression of LAT1 and 4F2hc transcripts with LAT1 binding partners in DRG and spinal cord.** Indicated gene expression values were extracted from the mousebrain.org database and then normalized to the maximum expression value for each gene for better visualization. (**A**) Scatter plot showing expression of Slc7a5 and Slc3a2 in DRG versus spinal cord. For this graph only, expression in the spinal cord was normalized to the maximum expression value in DRG. Gene expression for both was higher in DRG compared to spinal cord (Mann-Whitney, p < 0.0001). (**B**) Heatmap showing the normalized expression of Slc7a5 and Slc3a2 in DRG neuronal classes with functionally related genes Scn9a, Kcna1, Kcna2 and Cacna2d1. The neuronal markers Calca, P2rx3 and Nefh are shown to identify peptidergic, non-peptidergic and proprioceptor neurons respectively. (**C**) Heatmap showing the normalized expression of Slc7a5 and Slc3a2 in spinal cord neuronal classes with the functionally related genes Kcna1, Kcna2 and Cacna2d1. The markers of excitatory (Slc17a6) and inhibitory (Slc32a1) transmission are shown and the main neurotransmitter indicated according to the annotations curated from the database. Slc7a5: Large neutral amino acids transporter small subunit 1 (LAT1); Slc3a2: 4F2 cell-surface antigen heavy chain (4F2hc); Scn9a: Sodium channel protein type 9 subunit alpha (Nav1.7); Kcna1: Potassium voltage-gated channel subfamily A member 1 (Kv1.1); Kcna2: Potassium voltage-gated channel subfamily A member 2 (Kv1.2); Cacna2d1: Voltage-dependent calcium channel subunit alpha-2/delta-1 (α2δ-1); Calca: Calcitonin gene-related peptide 1 (CGRP); P2rx3: P2X purinoceptor 3 (P2X3R); Nefh: Neurofilament heavy polypeptide (NF200); Slc17a6: Vesicular glutamate transporter 2 (VGLUT2); Slc32a1: Vesicular inhibitory amino acid transporter (VGAT).

The expression of LAT transporter has been reported in the sensory system (Toyooka et al., 2008, Poncet et al., 2020). At embryonic day 9.5 (E9.5) of development, high levels of SLC7A5 transcripts were localised in the spinal cord, most strongly expressed in the spinal dorsal horn (SDH) (Poncet et al., 2020). Similarly, at E10.5 high expression of this transporter was found in neural crest (Poncet et al., 2020), which gives rise to the dorsal root ganglia (DRG) (Kasemeier-Kulesa et al., 2005). Databases of single cell RNA sequencing data compiled by the Linnarsson Lab at the Karolinska Institute allow the visualization of gene expression in different cell types (Zeisel et al., 2018). Such data showed that both LAT1 (slc7a5) and 4F2hc (slc3a2) expression is higher in DRG neurons compared to spinal cord (Fig. 1**A**) with levels in the dorsal horn of the spinal cord (not shown) being below the level of detection (Haring et al., 2018). In DRGs, LAT1 expression is enriched in Calcitonin Gene-Related Peptide (CGRP) positive peptidergic C-fibers and in NF200-positive myelinated Aδ fibers (proprioceptors) but is also expressed in non-peptidergic (P2X3R positive) DRG neurons (Fig. 1**B**). 4F2hc expression is enriched in non-peptidergic DRG neurons but low in other subclasses within this dataset (Fig. 1**B**). While significantly lower in the spinal cord, LAT1 expression is detected in 3 classes of inhibitory neurons and one class of excitatory neuron. 4F2hc expression can also be found in the same neuronal classes. These inhibitory neurons (INH1-3) are annotated as dorsal horn neurons located in the lamina 2–3, 6 and using γ-aminobutyric acid (GABA) and glycine as main neurotransmitters. The excitatory subclass of spinal neurons expressing LAT1 and 4F2hc is annotated as located in the dorsal horn lamina 4–6; these neurons use glutamate as their main neurotransmitter. Together these transcriptomics data indicate that in DRG neurons, LAT1 and 4F2hc are expressed on nociceptors and may have a role in nociceptive signal transmission. In the spinal cord, there is a low expression of the transporters in inhibitory neurons and in one particular class of excitatory neurons which may indicate a role in tonic inhibitory transmission in the spinal dorsal horn. These conclusions are limited by the absence, to this date, of a single cell RNA-seq study in the context of chronic neuropathic pain. At the protein level, the expression of both LAT1 and 4F2hc was detected in the spinal cord, with 4F2hc being the most abundant protein (Toyooka et al., 2008). Thus, the presence of LAT1 and its heterodimeric partner 4F2hc in nociceptive pathways suggests a possible involvement in pain signaling.

## 3 Regulation of LAT1 by the Wnt/β-catenin signaling pathway

Examination of the promoter region of SLC7A5 revealed that the transcription of this transporter depends on Wnt/β-catenin signaling pathway (Poncet et al., 2020). By using the eukaryotic promoter database and the MatInspector program (Cartharius et al., 2005), two putative lymphoid enhancer factor 1 (LEF1) and T-cell factor 7 (TCF7L2) sites upstream of the mouse SLC7A5 transcription start site were found (Poncet et al., 2020). This finding supported the possibility that SLC7A5 was a target of this pathway since β-catenin serves as a transcriptional coactivator of TCF/LEF family of transcription factors (Chen et al., 2006, Fuerer et al., 2008). In the same study, after exposing embryo trunk explants to Wnt secretion inhibitor Wnt-c59, it was found that SLC7A5′s expression was attenuated. Moreover, in embryos in which β-catenin was lost from the developing body axis, SLC7A5′s transcripts were absent in the neural tube compared to the littermate controls (Poncet et al., 2020), thus suggesting that Wnt/β-catenin signalling is required for SLC7A5′s transcription.

The Wnt signaling pathway contributes to the pathogenesis of inflammatory and neuropathic pain in rodents (Shi et al., 2012, Zhang et al., 2013b, Zhao and Yang, 2018). Nerve injury and bone cancer caused upregulation of Wnt/frizzled/β-catenin expression and signaling in DRG neurons, SDH neurons and astrocytes (Shi et al., 2012, Zhang et al., 2013b, Zhao and Yang, 2018). These Wnt signaling proteins are also up-regulated in the SDH after hind-paw injection of capsaicin, intrathecal injection of HIV-gp120 protein (Shi et al., 2012), as well as in a multiple sclerosis mouse model which develops chronic pain (Yuan et al., 2012). Further, intrathecal injection of Wnt production inhibitor IWP-2 into rodents effectively relieved pain behaviors (Zhang et al., 2013b, Zhao and Yang, 2018) and diminished the expression of Wnt/frizzled/β-catenin and reduced synaptic activity (Zhao and Yang, 2018). Recruitment of Wnt signaling stimulated the production of the proinflammatory cytokines interleukin 18 (IL-18) and tumor necrosis factor -alpha (TNF-α), as well as the N-methyl-D-aspartate receptor subunit NR2B in the spinal cord (Zhang et al., 2013b), which play a crucial role in pain pathways. These findings suggest that LAT1′s transcriptional regulation by Wnt’s signalling pathway might be a crucial mechanism underlying the pathogenesis of inflammation and neuropathic pain.

### 3.1 LAT1 links to neuropathic pain, inflammation, hypoxia and cancer

#### 3.1.1 Neuropathic pain

So far, there is only limited evidence of a possible role of LAT1 in neuropathic pain. Following a traumatic contusion injury in the rat spinal cord, increased immunoreactivity for LAT1 was detected on capillary endothelia in the region adjacent to the injury epicenter compared to the sham operated control (Toyooka et al., 2008). In contrast, immunoreactivity for 4F2hc was unchanged after injury. The results of western blot analysis from a 10-mm spinal cord segment containing the injury epicenter were consistent with those of immunohistochemistry (Toyooka et al., 2008). While in this study the paw withdrawal behavior to mechanical or thermal stimuli was not evaluated, it is well known that injury to the spinal cord results in neuropathic pain (Shiao and Lee-Kubli, 2018). Although the role of this protein in neuropathic pain has not been further investigated, its upregulation suggests that it may be a relevant target for future studies.

#### 3.1.2 Inflammation

LAT1 facilitates the influx of large neutral amino acids into T-cell-receptor (TCR)-activated T-cells and in doing so, promotes metabolic reprogramming necessary for T-cell activation and differentiation (Powell, 2013, Sinclair et al., 2013). The role of T-cells in chronic neuropathic and inflammatory pain states is an area of active research (Laumet et al., 2019). T-cells contribute to the generation and maintenance of neuropathic pain by infiltrating into the nervous system days to weeks post-nerve injury (Moalem et al., 2004, Agarwal et al., 2018). First, they infiltrate the site of injury and the spinal nerve, then the DRG, and finally the SDH (Moalem et al., 2004). Likewise, during inflammatory pain modeled by injection of Complete Freund’s adjuvant, formalin, or other inflammatory agents into the paw, increased infiltration of T-cell has been reported (Ghasemlou et al., 2015). It is worth recalling that DRG from naïve animals contain a low number of both CD4 + and CD8 + T-cells (Landis et al., 2012, Krukowski et al., 2016). However, after nerve injury, the T cells invading the DRG are mostly CD4+ (McLachlan and Hu, 2014). In this context, T-cells are barely detectable in the spinal cord of naïve animals. In response to spinal nerve injury, several studies have observed the presence of CD4 + T-cells in the SDH (Cao and DeLeo, 2008, Costigan et al., 2009). Thus, T-cell infiltration is a major contributor to neuropathic pain-like hypersensitivity.

Given the crucial role of LAT1 (Powell, 2013, Sinclair et al., 2013) and 4F2hc (Cantor et al., 2011) in T-cells, it is likely that LAT1-4F2hc complex could play an important role in chronic pain pathways related to T-cells. Indeed, it has already been shown that LAT1 is relevant in mediating inflammation associated with psoriasis, a hyperproliferative skin disorder (Cibrian et al., 2020). The expression of LAT1 is enhanced in many cancer cells (Hayase et al., 2017), including malignant skin lesions (Shimizu et al., 2015). Interestingly, increased transcriptional levels of SLC7A5 were detected in skin samples of patients with psoriasis (Cibrian et al., 2016). Similarly, LAT1′s expression is augmented in keratinocytes and skin infiltrating lymphocytes of psoriatic lesions in human and mice (Cibrian et al., 2020). Pharmacological inhibition of LAT1 with JPH203 has an anti-inflammatory effect and might contribute to prevention of psoriasis (Cibrian et al., 2020). Likewise, specific deletion of LAT1 in gamma delta (ɣδ) and CD4 + T-cells controls the inflammatory response in a model of skin inflammation induced by imiquimod (Cibrian et al., 2020).

B-cells also contribute to tissue inflammation (Griss et al., 2019). Recent studies have reported that in imiquimod (toll-like receptor 9 (TLR9) ligand)-stimulated human B-cells, there is enhanced expression of LAT1-4F2hc in the plasma membrane and an increased influx of L-leucine (Torigoe et al., 2019). This influx is suppressed by pre-treatment with 2-aminobicyclo-(2,2,1)-heptane-2-carboxylic acid (BCH**)** (Torigoe et al., 2019), a competitive inhibitor of this transporter (Hendrich et al., 2008), indicating that LAT1 is mainly responsible for the enhanced transport of L-leucine. It is worth noting that upregulation of LAT1′s expression induces activation of serine/threonine kinase mammalian target of rapamycin (mTOR) in T-cells (Cibrian et al., 2020) and the mTOR complex 1 (mTORC1) in B-cells (Torigoe et al., 2019) and both events are coupled to the influx of L-leucine through LAT1 (Schriever et al., 2013, Sinclair et al., 2013). Abrogation of L-leucine’s influx by BCH leads to a decrease of mTORC1 activity in B-cells (Torigoe et al., 2019). mTOR controls T-cell differentiation (Finlay et al., 2012) and, together with LAT1, controls inflammatory responses in the imiquimod-induced psoriasis model (Cibrian et al., 2020). mTORC1 signaling allows B-cells to migrate into inflammatory sites and to enter a metabolic reprogramming state that enables biosynthesis of immunoglobulins and cytokines (Torigoe et al., 2019).

Recent studies have shown that the mTORC1 signaling axis is upregulated in the spinal cord in chronic pain conditions (Geranton et al., 2009, Torigoe et al., 2019). Inhibition of this complex with intrathecal administration of rapamycin effectively alleviated inflammatory responses (Price et al., 2007, Li et al., 2019) and neuropathic pain (Geranton et al., 2009, Zhang et al., 2013a). The mechanisms by which rapamycin alleviates pain hypersensitivity in a chronic neuropathic pain model was reported by Zhang and colleagues (Zhang et al., 2013a). Rapamycin attenuates the expression of postsynaptic density 95 (PSD95) (Zhang et al., 2013a), a scaffold protein that is abundantly enriched in the PSD of excitatory synapses (Kim et al., 2007). Rapamycin inhibits PSD95′s interactions with α-amino-3-hydroxy-5-methyl-4-isoxazolepropionic acid (AMPA) and NMDA receptor subunits (Zhang et al., 2013a), receptors that have been proven to be crucial components in pain signaling (Liu and Salter, 2010). This suggests that mTOR is an important kinase involved in chronic pain, since its blockade attenuates nerve injury-induced changes in excitability (Geranton et al., 2009). Overall, it appears that targeting the LAT1-mTOR axis may offer opportunities to intervene to prevent or control chronic neuropathic pain and inflammation. Put another way, the Wnt/B-catenin pathway could drive increases in LAT1 expression and subsequent L-leucine influx, which could increase mTOR in T-cells to mediate chronic neuropathic and inflammatory pain

## 4 Hypoxia and cancer

Reduced oxygen availability (hypoxia) affects LAT1′s expression through hypoxia-inducible factor 2α (HIF-2α) (Elorza et al., 2012, Onishi et al., 2019). HIF-2α is a transcriptional factor that promotes adaptation to hypoxic/ischemic stress in response to low tissue oxygenation (Simon, 2016). HIF-2α possesses proliferative and tumor promoting properties (Raval et al., 2005, Gordan et al., 2007). In the differentiated neuroblastoma cells (Neuro2A) exposed to hypoxia, the expression of LAT1 is significantly increased (Onishi et al., 2019). In the same study, when cells were retrovirally infected using short hairpin RNA (shRNA) for HIF-2α, hypoxia-induced upregulation of the expression of LAT1 was completely blocked (Onishi et al., 2019). Surprisingly, hypoxia induces not only LAT1′s expression but also mTORC1′s activation in differentiated neuronal cells (Onishi et al., 2019). Another study documented that in tumor cells deficient of von Hippel-Lindau (VHL) factor, a component in the E3 ubiquitin ligase complex that marks HIF-2α for degradation, activation of the HIF-2α pathway increases mTORC1′s activity by upregulating the expression of LAT1 (Elorza et al., 2012). Of relevance here, it was demonstrated that HIF-2α binds to the SLC7A5 proximal promoter through its DNA binding domain, and it therefore contributes to SLC7A5′s gene expression in tumor cells (Elorza et al., 2012), and under hypoxic conditions (Onishi et al., 2019).

Although the role of HIF-2α in neuropathic pain has not yet been studied, peripheral nerve injury causes persistent endoneurial hypoxia (Lim et al., 2015). Notably, expression of the related HIF-1α was observed at the injury site as early as 2 h after injury and persisted until at least day 28 (Lim et al., 2015). When HIF-1α was specifically deleted in mice DRG neurons, mice were more sensitive to noxious heat and cold pain stimulation in inflammatory and neuropathic pain models (Kanngiesser et al., 2014). Based on this evidence, it is therefore likely that upregulation of the HIF-2α/LAT1 axis also plays a relevant role in pain, since it is capable of activating mTORC1 through the regulation of LAT1′s expression.

### 4.1 Interactions partners of SLC7A5

Among other interactions, LAT1 has been reported to interact with channels with demonstrable links to pain, particularly a subtype of the voltage-gated potassium (Kv1.1) (Baronas et al., 2018) and the voltage-gated sodium channels Nav1.7 (Kanellopoulos et al., 2018).

#### 4.1.1 Kv channels

Kv1.1 and Kv1.2 channels are localized in the SDH (Coleman et al., 1999, Karimi-Abdolrezaee et al., 2004, Utsunomiya et al., 2008) and in DRG neurons (Ishikawa et al., 1999, Glazebrook et al., 2002). K^+^ channel function is fundamental for sensory neuron activity during pain signalling (Glazebrook et al., 2002, Gonzalez et al., 2017). As increased neuronal excitability is a hallmark of pathological pain, opening of these channels creates a hyperpolarizing K^+^ efflux across the cell membrane, inhibiting excitability (Glazebrook et al., 2002). However, following axonal injury, Kv1.1 and Kv1.2 immunoreactivity in DRG neurons decreases (Ishikawa et al., 1999, Fan et al., 2014). α-dendrotoxin, a selective inhibitor of these channels (Harvey, 2001), induces repetitive action potential firing of sensory neurons (Hall et al., 1994, Glazebrook et al., 2002). In addition, Kv1.1 knockout mice display hyperalgesia, consistent with an important role for these channels in nociception (Clark and Tempel, 1998). In this context, recent studies have reported that LAT1 regulates Kv1.1 (Sharmin et al., 2020) and KV1.2 (Baronas et al., 2018) function. Co-expression of LAT1 and Kv1.1 or Kv1.2 in mouse leukocyte tyrosine kinase (ltk)-deficient fibroblast cells, markedly decreased Kv1.1 (Sharmin et al., 2020) and Kv1.2 (Baronas et al., 2018, Lamothe and Kurata, 2020) current density. Similarly, LAT1 reduced Kv1.2 total expression (Baronas et al., 2018, Lamothe and Kurata, 2020). Interestingly, Kv1.2 is not affected by co-expression with LAT2 (SLC7A6), a transporter that also heterodimerizes with SLC3A2 (Pineda et al., 1999). In contrast, LAT1 did not influence Kv1.5 (Baronas et al., 2018), suggesting that Kv1.1 and Kv1.2 regulation is selective for LAT1. Moreover, LAT1 caused KV1.2 channels to accumulate in a non-conducting state that requires hyperpolarized holding voltages (~–120 mV or more negative) to relieve the current suppression (Baronas et al., 2018). In the presence of LAT1, Kv1.1 exhibits prominent disinhibition when membrane voltage was held at –120 mV. This effect was attenuated by shRNA knockdown of endogenous LAT1 (Sharmin et al., 2020). Under physiological conditions, these extreme voltages are never reached, therefore this molecular complex would act as a trap that could alter excitability by silencing Kv channels (Baronas et al., 2018). The canonical accessory subunit of Kv1.2, KVβ, promotes N-type inactivation (Rettig et al., 1994), cell surface expression (Gu et al., 2003), and function (Lamothe and Kurata, 2020) of the channel. It has been reported that the degree of inactivation of this channel is greater in the presence of LAT1 (Lamothe and Kurata, 2020), suggesting that the N-terminal inactivation particle of KVβ binds more favorably to the pore of Kv1.2 when LAT1 is present. Consequently, recovery of the channel from inactivation is significantly delayed (Lamothe and Kurata, 2020). In DRG neurons LAT1 and Kv1.1/Kv1.2 are co-expressed in proprioceptors (NF1-3 subclasses) while in spinal cord their co-expression can be seen in the INH2 subclass (Fig. 1**B-C**). It still remains unclear whether Kv1.1 or Kv1.2 are regulated by a direct interaction with LAT1. Nonetheless, it is possible that the downregulation of Kv1.1 and Kv1.2 by LAT1 is involved in neuropathic pain conditions, and that this may be the cause of an increased excitability in pain.

#### 4.1.2 NaV1.7 channels

**Fig. 2:**
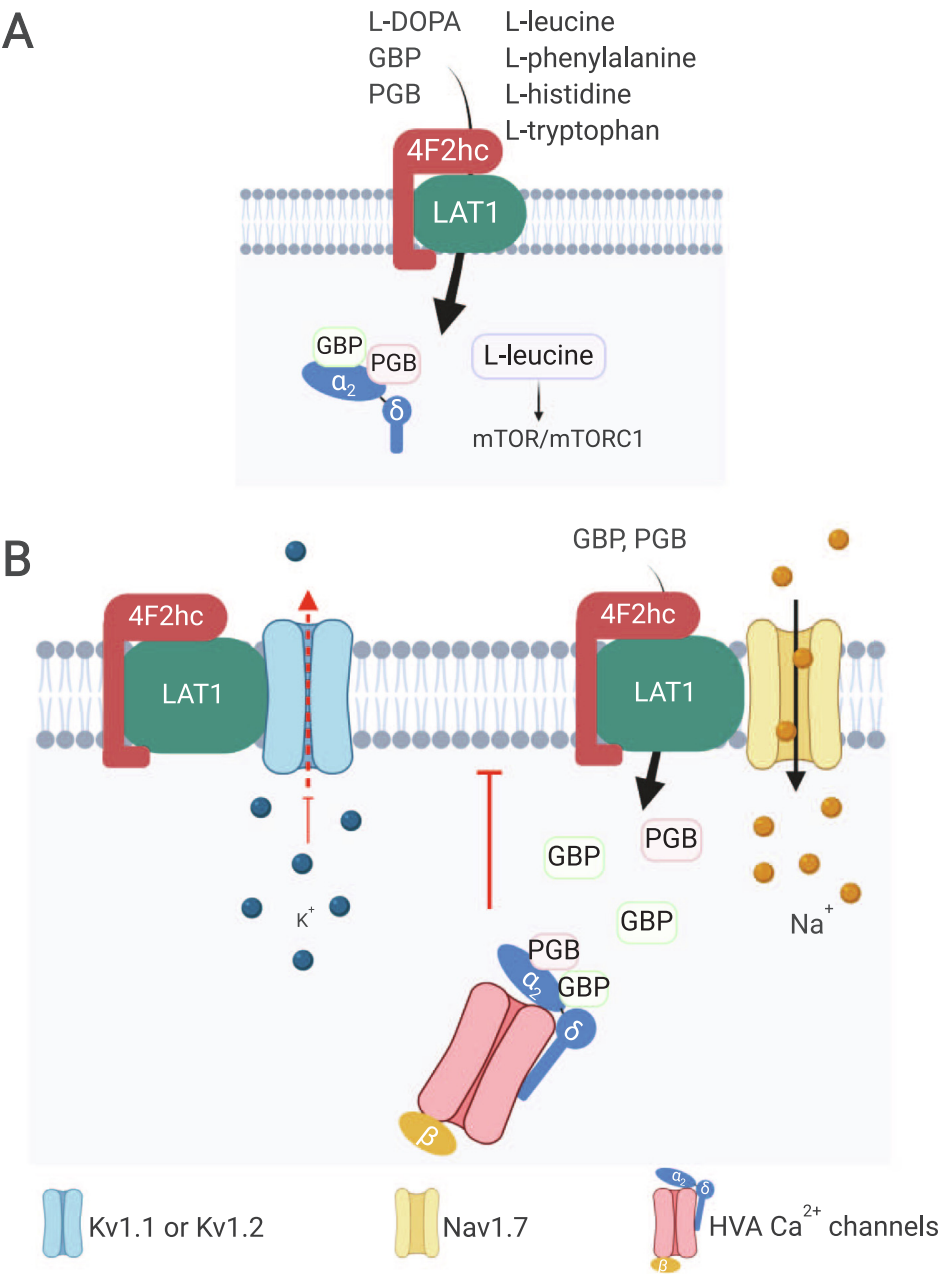
**The LAT1-4F2hc complex, its substrates and interactions with voltage-gated ion channels.** (**A**) LAT1 mediates the influx of gabapentin (GBP) and pregabalin (PGB), which have been reported to bind to the α2δ-1 auxiliary subunits of high voltage-activated (HVA) Ca^2+^ channels. LAT1 also regulates mTOR/mTORC1 activity by controlling the influx of L-leucine. (**B**) The interaction of LAT1 with Kv1.1 or Kv1.2 causes the accumulation of these channels in a non-conducting state. LAT1 exists in a complex with Nav1.7 but whether this influences channel activity is unknown. LAT1-mediated influx of GBP and PGB allows the interaction of these drugs with α2δ-1 subunit which then inhibits the trafficking of HVA Ca^2+^ channels to the plasma membrane.

Kanellopoulos and colleagues have demonstrated that LAT1 interacts with the sodium channel Nav1.7 (Kanellopoulos et al., 2018) (Fig. 2). This channel is highly expressed in DRG neurons and in the presynaptic terminals in the SDH (Black et al., 2012). Nav1.7 is a tetrodotoxin-sensitive (TTX-S) sodium channel that produces a fast-activating, fast-inactivating current (Klugbauer et al., 1995), and is considered a threshold channel (Alexandrou et al., 2016). Increased Nav1.7 expression has been reported in multiple animal models of inflammatory (Black et al., 2004, Liang et al., 2013) and neuropathic pain (Hong et al., 2004, Li et al., 2018), in DRG neurons (Hong et al., 2004, Liang et al., 2013, Li et al., 2018), and in the SDH (Li et al., 2018). Nav1.7-dependent hyperexcitability of primary afferent sensory neurons is upregulated (Hong et al., 2004, Zhang and Dougherty, 2014). This channel has been implicated in rare human genetic conditions involving Nav1.7 mutations, demonstrating its role in pain pathways (Yang et al., 2004, Cox et al., 2006, Fertleman et al., 2006). Therefore, Nav1.7 has been catalogued as a molecular target in the battle against chronic pain.

A previous report identified LAT1 as a novel Nav1.7 channel-interacting protein from an epitope-tagged Nav1.7 mouse (Kanellopoulos et al., 2018). This protein–protein interaction was validated using a co-immunoprecipitation assays by transfecting LAT1 into HEK-293 cell line stably expressing TAP-tagged Nav1.7 (Kanellopoulos et al., 2018). The interaction of this channel with other proteins such as the post-translationally modified collapsin response mediator protein 2 (CRMP2), is essential for the trafficking of functional channels to the plasma membrane in pain signaling (Dustrude et al., 2016, Chew et al., 2019). Recently, Zhang’s group revealed that fibroblast growth factor 13 regulates heat nociception by interacting with Nav1.7 and maintaining the membrane localization of the channel (Yang et al., 2017). In DRG neurons, LAT1 and Nav1.7 (Scn9a) are coexpressed in non-peptidergic neurons (Fig. 1**B**). Hence, it is possible that LAT1 may also be involved in the upregulation of Nav1.7 channel function in neuropathic pain. However, future experiments are needed to determine the functional significance of the LAT1-Nav1.7 interaction in this regard.

## 5 LAT1 involvement in synaptic dysfunction in neurological disorders

Anomalies in genes encoding LAT1 and other large amino acid transporters have been linked to autism (Tarlungeanu et al., 2016, Cascio et al., 2020) and schizophrenia (Comasco et al., 2016). Abnormalities in the ratio of excitatory to inhibitory activity (E-I imbalance) in neuronal circuits is a common feature of autism and schizophrenia spectrum disorders (Canitano and Pallagrosi, 2017). In this context, it is possible that LAT1 may play a role in governing this imbalance. Indeed, E-I imbalance also occurs in chronic neuropathic pain states (Alles and Smith, 2018). Excitatory synaptic processes are enhanced, and inhibitory processes are attenuated (West et al., 2015). However, the precise role of LAT1 in regulating E-I imbalance in nociception has yet to be determined.

Strikingly, γ-aminobutyric acid (GABA)-ergic and glutamatergic signaling are regulated by mTOR pathway (Weston et al., 2012). Since L-leucine induces activation of mTORC1 (Schriever et al., 2013, Wolfson et al., 2016), and because its signaling pathway is upregulated in pain conditions (Khoutorsky and Price, 2018), it is reasonable to propose that LAT1-mTORC1 axis might be involved in this imbalance.

## 6 Gabapentinoid transport

The first-line neuropathic pain drugs, the gabapentinoids, enter neurons via L-type amino acid transporter (Su et al., 1995). Their effectiveness depends on access to the α2δ-1 auxiliary subunit of high voltage-activated (HVA) Ca^2+^ channels (Bauer et al., 2009). This subunit enhances the trafficking of functional channels to the plasma membrane (Felix et al., 1997, Canti et al., 2005), thus increasing Ca^2+^ currents (Felix et al., 1997). Likewise, α2δ-1 has been found to be involved in mammalian excitatory synaptogenesis through binding to extracellular matrix proteins of the thrombospondin family, inducing excitatory synapse formation (Eroglu et al., 2009). Previous studies have documented that peripheral nerve injury enhances the expression of α2δ-1 in the DRG (Newton et al., 2001, Luo et al., 2002, Gomez et al., 2019) and SDH (Luo et al., 2002, Li et al., 2004, Boroujerdi et al., 2011). This increased expression correlates with neuropathic pain development and maintenance (Luo et al., 2002, Li et al., 2004, Boroujerdi et al., 2011).

An important therapeutic class of drugs, GBP and PGB exert their analgesic action by binding to α2δ-1 subunits (Field et al., 2006, Lau et al., 2017). It is thought that GBP displaces an endogenous ligand (L-leucine) that is a positive modulator of the subunits (Felix et al., 2013), and that it inhibits post-Golgi forward trafficking of α2δ-1 (Bauer et al., 2010). This results in an inhibition of Ca^2+^ currents (Field et al., 2006) by impairing the ability of the subunits to enhance trafficking of Ca^2+^ channels to the DRG plasma membrane (Hendrich et al., 2008) and their presynaptic terminals in the SDH (Bauer et al., 2009).

GBP and PGB are transported into the neuronal cytoplasm via a L-type amino acid transporter (Su et al., 1995). The influx of gabapentinoid drugs was demonstrated to be mediated by LAT1 in brain endothelial cells and in HEK-293 cells stably expressing LAT1 (Dickens et al., 2013, Takahashi et al., 2018) (Fig. 2). LAT1 has been proposed to be responsible for the permeation of amino acid-related drugs through the plasma membrane (Uchino et al., 2002). In line with this evidence, gabapentin can resemble a L-form of large neutral amino acids (Su et al., 1995), giving it strong structural similarity to endogenous substrates of LAT1 (Singh and Ecker, 2018). On the other hand, short interfering RNA (siRNA) mediated suppression of LAT1 (Dickens et al., 2013, Takahashi et al., 2018) provided evidence that GBP is transported specifically by this transporter since its uptake was significantly reduced compared to LAT2 targeted siRNA (Dickens et al., 2013). Importantly, BCH inhibits gabapentinoid transport (Su et al., 2005, Akanuma et al., 2018) and prevents the effect of GBP on HVA Ca^2+^ channels (Hendrich et al., 2008, Biggs et al., 2014). Work from Biggs and colleagues has demonstrated that 300 μM of BCH, can prevent a GBP- or PGB-mediated attenuation in an evoked increase neuronal excitability in the spinal cord (Biggs et al., 2014). LAT1 and α2δ-1 present with a high level of co-expression in DRG neurons especially in the PEP2 (peptidergic C-fiber) class (Fig. 1**B**) as well as in the GLU1 subclass in the spinal dorsal horn (Fig. 1**C**). Therefore, the activity of LAT1 is necessary for GBP and PGB actions on preventing neuronal hyperexcitability and therefore alleviating pain.

## 7 Conclusions

The evidence presented in this review suggests that LAT1 activity regulates a number of ion channels and auxiliary subunits. Based on the still developing literature, we posit that LAT is a protein that may be a relevant contributor in chronic pain conditions either by interacting with potassium and sodium ion channels, by inducing the activation of mTOR signaling pathway, or by regulation of the E-I imbalance. The relative contributions of LAT1 within the DRG and DH in the context of pain are not yet fully known. However, evidence presented here linking LAT1 expression, function, and downstream signalling pathways to pain suggests that it may be a viable new target in chronic pain.

## Declaration of Competing Interest

R. Khanna is the co-founder of Regulonix LLC, a company developing non-opioids drugs for chronic pain. In addition, R. Khanna has patents US10287334 and US10441586 issued to Regulonix LLC. The remaining authors declare that they have no known competing financial interests or personal relationships that could have appeared to influence the work reported in this paper.
